# Response of Fecal Bacterial Flora to the Exposure of Fumonisin B1 in BALB/c Mice

**DOI:** 10.3390/toxins13090612

**Published:** 2021-08-31

**Authors:** Fan Zhang, Zhiwei Chen, Lin Jiang, Zihan Chen, Hua Sun

**Affiliations:** State Key Laboratory of Bioactive Substance and Function of Natural Medicines, Institute of Materia Medica, Chinese Academy of Medical Sciences & Peking Union Medical College, 1# Xiannongtan Street, Xicheng District, Beijing 100050, China; AFUN008034@163.com (F.Z.); chenzw@imm.ac.cn (Z.C.); gillianjiang@imm.ac.cn (L.J.); chenzihan@imm.ac.cn (Z.C.)

**Keywords:** fumonisin B1, BALB/c mice, fecal bacterial flora, 16S rRNA sequencing

## Abstract

Fumonisins are a kind of mycotoxin that has harmful influence on the health of humans and animals. Although some research studies associated with fumonisins have been reported, the regulatory limits of fumonisins are imperfect, and the effects of fumonisins on fecal bacterial flora of mice have not been suggested. In this study, in order to investigate the effects of fumonisin B1 (FB1) on fecal bacterial flora, BALB/c mice were randomly divided into seven groups, which were fed intragastrically with 0 mg/kg, 0.018 mg/kg, 0.054 mg/kg, 0.162 mg/kg, 0.486 mg/kg, 1.458 mg/kg and 4.374 mg/kg of FB1 solutions, once a day for 8 weeks. Subsequently, feces were collected for analysis of microflora. The V3-V4 16S rRNA of fecal bacterial flora was sequenced using the Illumina MiSeq platform. The results revealed that fecal bacterial flora of mice treated with FB1 presented high diversity. Additionally, the composition of fecal bacterial flora of FB1 exposure groups showed marked differences from that of the control group, especially for the genus types including Alloprevotella, Prevotellaceae_NK3B31_group, Rikenellaceae_RC9_gut_group, Parabacteroides and phylum types including Cyanobacteria. In conclusion, our data indicate that FB1 alters the diversity and composition of fecal microbiota in mice. Moreover, the minimum dose of FB1 exposure also causes changes in fecal microbiota to some extent. This study is the first to focus on the dose-related effect of FB1 exposure on fecal microbiota in rodent animals and gives references to the regulatory doses of fumonisins for better protection of human and animal health.

## 1. Introduction

Evidence has suggested that the intestinal bacterial flora plays an important role in maintaining the health of the body [1]. This community consists of trillions of bacteria, which are beneficial to nutrition collection, immunity intrusion, barrier function, and energy regulation [2,3,4]. However, stimulation or interference including physical destruction, xenobiotics, host factors and antimicrobial drugs, may cause changes in the numbers or species of intestinal bacterial flora [5,6], which result in diseases by affecting the proinflammatory activity [7], metabolism [8] and modulation of gut hormones [9]. Mycotoxins are secondary metabolites produced by fungi, which are capable of causing noxious effects on humans and animals [10,11]. Increased investigations on mycotoxins, such as aflatoxin B1 [12,13], deoxynivalenol [14,15], zearalenone [16], trichothecene [17], etc., have proved that exposure of mycotoxins could influence the diversity and composition of gut microbiome. Meanwhile, the intestinal bacterial flora has the ability to bind, degrade or transform mycotoxins [18,19]. Mycotoxins and intestinal bacterial flora may interact with each other.

Fumonisins are a group of mycotoxins produced by *Fusarium moniliforme* and other *Fusarium* species, which commonly contaminate corn, sorghum, related grains and even traditional Chinese medicines (TCM) throughout the world [20]. There are 28 fumonisin analogs that have been characterized since 1988 and they are separated into four main groups, identified as the fumonisin A, B, C and P series [21]. Among those many kinds of fumonisins known, fumonisin B1 (FB1) is the most abundantly produced member and the most toxic [22]. It has been proved that FB1 is neurotoxic [23], nephrotoxic, hepatotoxic [22], hepatocarcinogenic [24] and immunotoxic [25]. As a potential hazardous contaminant, FB1 has been shown to cause the production of equine leukoencephalomalacia (ELEM) and porcine pulmonary edema (PPE) [20,26]. It is also regarded as a high incidence of human esophageal cancer [26]. Contamination of food [27,28,29,30], feed [31,32,33] and TCM [34] with the fumonisins has been a more and more serious concern in our society. In recent years, the toxicity and the toxic mechanism of fumonisins has become a research hotspot following the aflatoxins. However, unlike aflatoxins, the toxicity of FB1 is still underestimated and the regulations associated with the guidance levels of fumonisins on food, feed or TCM are not currently exhaustive.

Previous investigations about FB1 mostly focused on the body organs’ toxicity and its carcinogenicity [35]. Some researchers are committed to finding advanced methods to detect the amount of FB1 [36,37], and some are attempting to transform and remove FB1 to decrease its toxicity [38]. Few studies pay attention to the effects of FB1 on gut bacterial flora. The only two articles found were about the effect of a single dose of FB1 on gut microbiota in weaned pigs [39] and broiler chickens [40], which suggested that FB1 could disturb the normal activities of bacterial flora. The effects of different levels of FB1 on gut bacterial flora in rodents have not been investigated so far. In this study, six different concentrations of FB1 solutions, which were set according to the current regulations throughout the world, were continuously administered to female BALB/c mice, which are more sensitive to the toxicity of FB1 [41,42,43]. The results provide further evidence about the influence of FB1 on the fecal bacterial flora, especially low levels of FB1 for long-term exposure. This article is expected to attract more attention to the toxicity of FB1, and also hope to give references to the guidance levels of fumonisins and put forward methods to reduce the toxicity through modulating the gut bacterial flora.

## 2. Results

### 2.1. Diversity of Fecal Bacterial Communities of Mice

**Figure 1:**
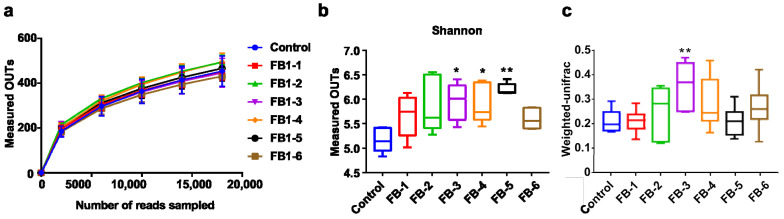
The diversity of the fecal bacterial community. (**a**) Rarefaction curve. (**b**) Alpha diversity revealed by the Shannon index. (**c**) Beta diversity revealed by weighted unifrac distance. * means *p* < 0.05, ** means *p* < 0.01 vs. control group.

**Figure 2:**
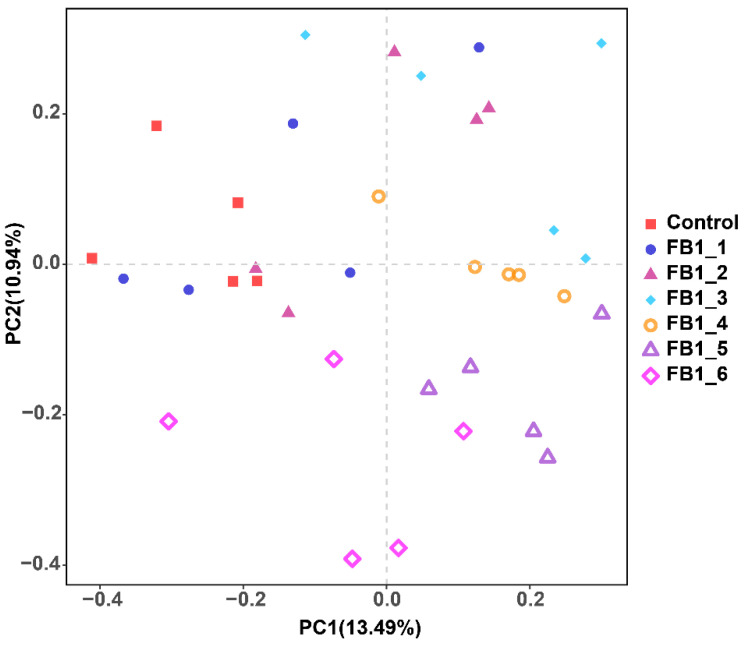
The PCA of the bacterial flora in groups.

The rarefaction cure revealed that the measured sequence number was reasonable for further analysis. All rarefaction cures of the seven groups plateaued, which indicated that the majority of sequences were involved in the analysis process for each group (Figure 1a). Alpha diversity was applied in analyzing the complexity of species diversity for each group. Based on alpha diversity analysis, the Shannon index was identified. After 8 weeks of exposure to FB1, the Shannon diversity index tended to be higher in feces from FB1-treated groups, especially in FB1-3, FB1-4 and FB1-5 groups, compared to those in the control group. Unusually, FB1-6 was slightly decreased relative to other groups but still increased compared with control group. These results suggested that FB1 exposure was accompanied by higher diversity in feces microbiota (Figure 1b). Beta diversity was used to evaluate differences in the species complexity in samples and was showed by a weighted unifrac index. Only the fecal bacterial flora of FB1-3 was significantly increased compared with the control group. Based on the abundance of every OTU (Figure 1c), the data distribution of groups was exhibited by PCA (Principal Component Analysis), which indicated that FB1-3, FB1-4, FB1-5 and FB1-6 groups were separated well from the control group (Figure 2).

### 2.2. Microbiota Profiles in Feces of Mice

**Figure 3:**
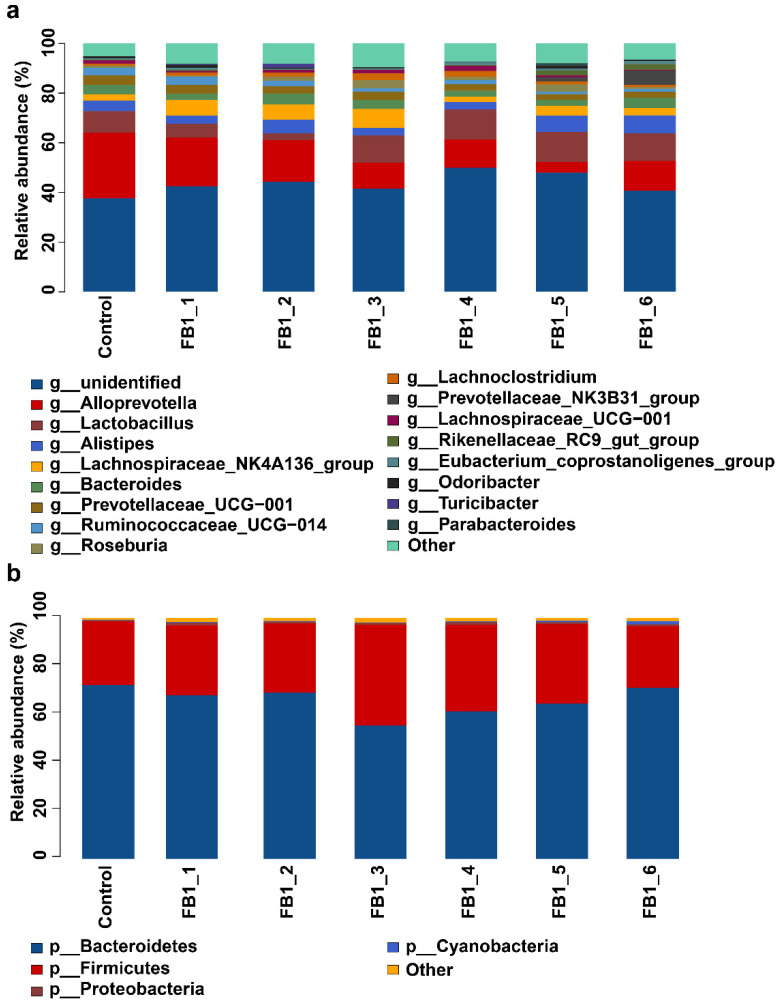
Column pictures of genus and phylum types and relative abundance of fecal bacterial flora. Others: Fecal bacterial flora with relative abundance < 1% were included as others. (**a**) Column pictures of genera; (**b**) column pictures of phyla.

**Figure 4:**
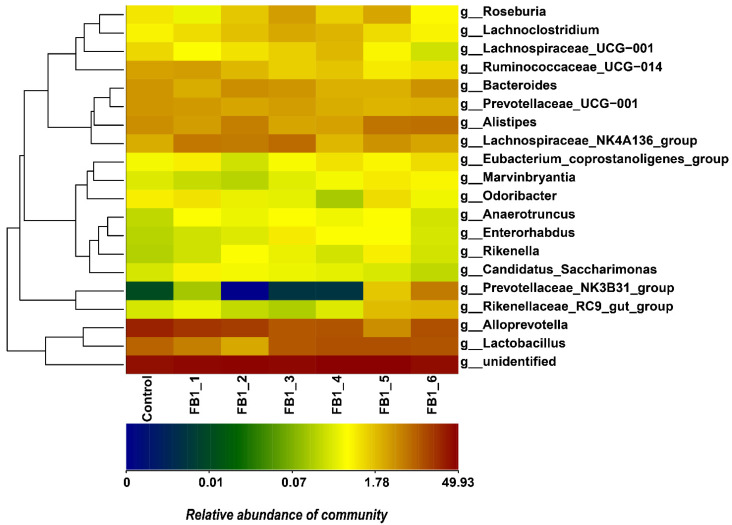
Heatmap of genus types and relative abundance of fecal bacterial flora. Others: Fecal bacterial flora with relative abundance < 1% were included as others.

**Figure 5:**
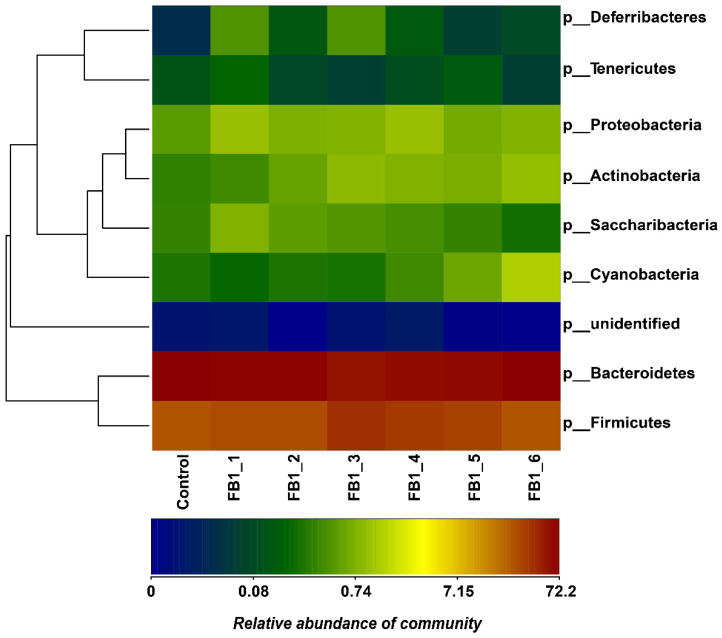
Heatmap of phylum types and relative abundance of fecal bacterial flora. Others: Fecal bacterial flora with relative abundance <1% were included as others.

The relative abundances of fecal bacteria in genus types were drawn as a column picture and a heatmap as shown in Figure 3, Figure 4 and Figure 5. The 10 most abundant genera were Alloprevotella, Lactobacillus, Alistipes, Lachnospiraceae_NK4A136_group, Bacteroides, Prevotellaceae_UCG-001, Ruminococcaceae_UCG-014, Roseburia, Lachnoclostridium and Prevotellaceae_NK3B31_group, respectively. Concerning the relative abundances of fecal bacteria in phylum types, it was obtained that the Bacteroides phylum was the most abundant one in the fecal microbiota from all groups. Firmicutes was the second most abundant phylum followed by Proteobacteria and Cyanobacteria. Furthermore, Cyanobacteria tended to be higher in all FB1-treated groups than in the control group. There were also another four phylum types with relatively lower abundance (<1%) in the heatmap: Actinobacteria, Saccharibacteria, Deferribacteria and tenericutes.

### 2.3. Phylogenetic Tree

**Figure 6:**
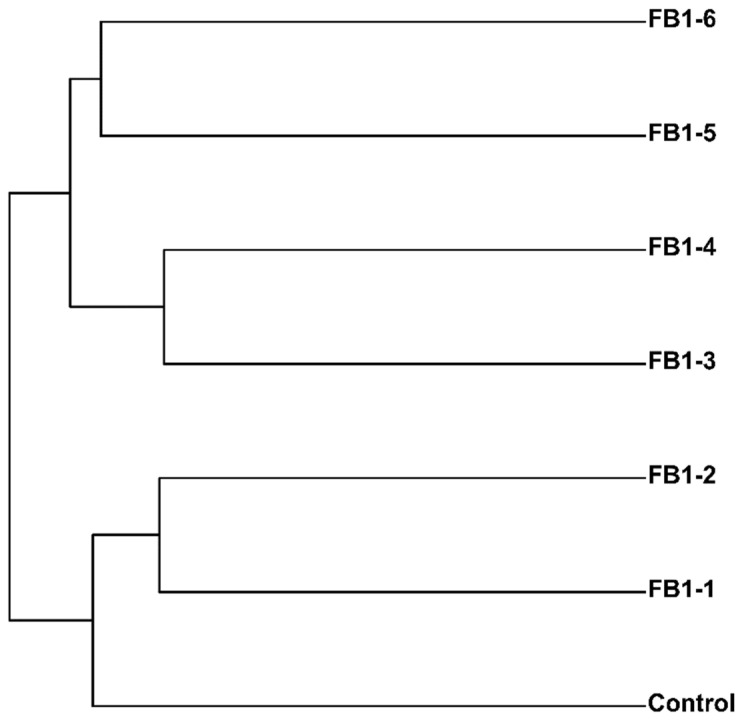
Phylogenetic tree obtained based on the hierarchical clustering analysis.

The phylogenetic tree was obtained based on the hierarchical clustering analysis according to the distance matrix in the beta diversity analysis (Figure 6). The fecal bacteria of the control and the low-dose groups including FB1-1 and FB1-2 were clustered together, which indicates that these three groups had a relatively close genetic relationship. In addition, FB1-3 and FB1-4, and FB1-5 and FB1-6 were clustered together, respectively, showing a close genetic relationship.

### 2.4. Shifts in Relative Abundance of Fecal Bacterial Flora

#### 2.4.1. Genera Performances

**Figure 7:**
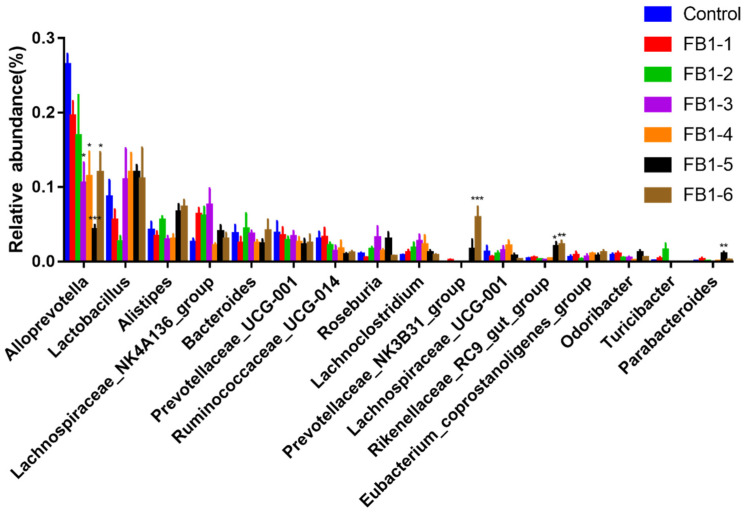
Difference in relative abundance of fecal bacterial flora between control and FB1 treatment groups at the genus level. * means *p* < 0.05, ** means *p* < 0.01, *** means *p <* 0.001 vs. control group.

As shown in Figure 7, compared with the control group, Alloprevotella decreased significantly in FB1-3, FB1-4, FB1-5 and FB1-6 groups. Prevotallaceae_UCG-001 and Ruminococcaceae_UCG-014 tended to decrease in FB1-exposed groups compared with the control group. In contrast, Prevotellaceae_NK3B31_group, Rikenellaceae_RC9_gut_group and Parabacteroides showed a significant increase in the FB1-5 group and the FB1-6 group. In addition, there was an interesting phenomenon that Lactobacillus and Alistipes decreased in lower doses first and increased in higher doses, while Lachnospiraceae_NK4A136_group, Lachnoclostridium and Lachnospiraceae_UCG-001 typically increased in lower doses and decreased in higher doses.

#### 2.4.2. Phyla Performances

**Figure 8:**
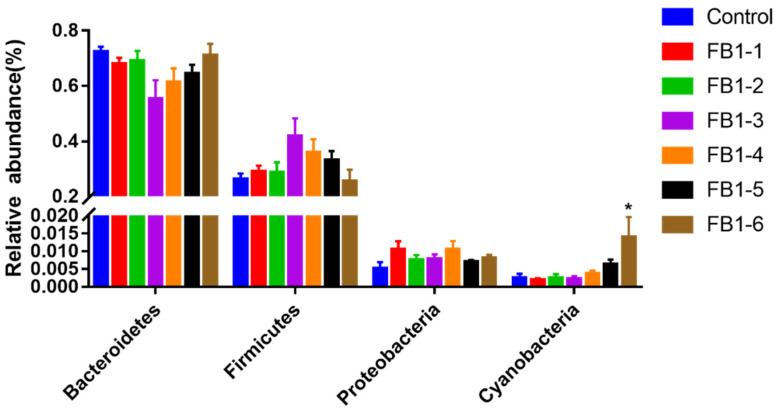
Difference in relative abundance of fecal bacterial flora between control and treatment groups at the phylum level. * means *p <* 0.05 vs. control group.

As for phyla types, Bacteroidetes and Firmicutes presented opposite tends (Figure 8). We determined that Bacteroidetes decreased in FB1-1, FB1-2 and FB1-3 groups compared to the control group and increased in FB1-4, FB1-5 and FB1-6 groups, while Firmicutes showed increases in FB1-1, FB1-2 and FB1-3 groups and decreases in FB1-4, FB1-5 and FB1-6 groups. For Proteobacteria, no evident change occurred in all FB1 exposure groups. The relative abundance of Cyanobacteria showed a significant increase in the FB1-6 group compared to the control group.

### 2.5. LEfSe Analysis

**Figure 9:**
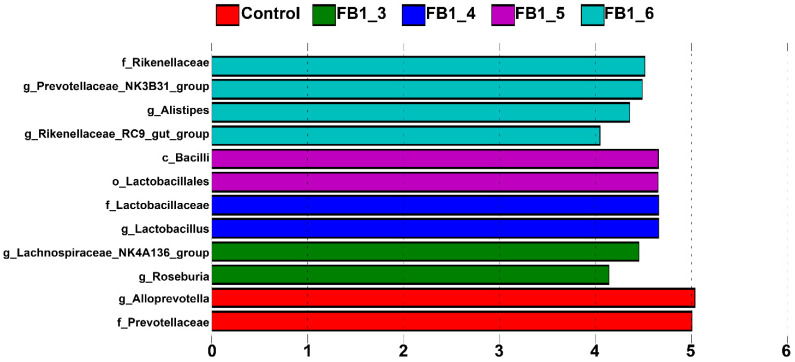
LEfSe comparison of fecal bacterial flora in seven groups.

LEfSe analysis was also performed with an LDA score of three to evaluate the community structure variance of bacterial flora between the seven groups. Compared with FB1 treatments, Figure 9 clearly showed that Alloprevotella remained the absolute predominant bacteria in the control group. There were six genera that had an obvious increase in FB1 treatments (FB1-3, FB1-4, FB1-5 and FB1-6 groups): Roseburia, Lachnospiraceae_NK4A136_group, Lactobacillus, Rikenellaceae_RC9_gut_group, Alistipes and Prevotellaceae_NK3B31_group. Additionally, there were no significant changes in FB1-1 and FB1-2 groups.

### 2.6. Microbial Function Prediction

**Figure 10:**
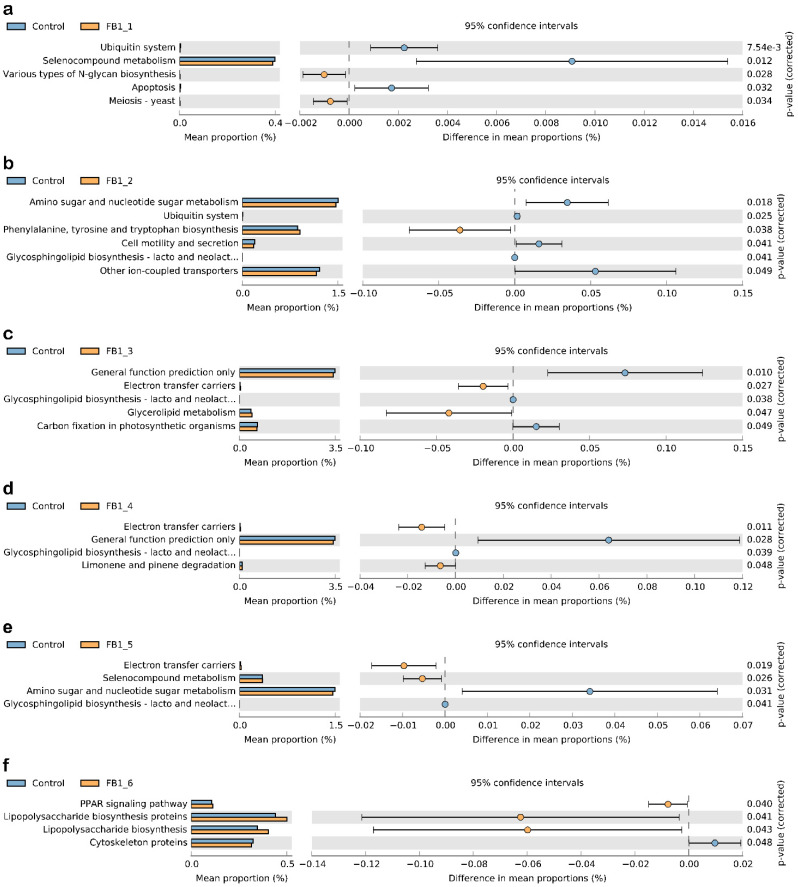
KEGG pathway analysis for fecal bacterial flora in seven groups. (**a**) Control group vs. FB1-1 group. (**b**) Control group vs. FB1-2 group. (**c**) Control group vs. FB1-3 group. (**d**) Control group vs. FB1-4 group. (**e**) Control group vs. FB1-5 group. (**f**) Control group vs. FB1-6 group.

In order to evaluate the effects of FB1 on the fecal bacterial flora of mice, the KEGG database was used to compare and predict the function and metabolic pathways based on the microbiota flora in these seven groups. Through comparing the control with each FB1-treated group, we made some interesting observations. Both FB1-1 and FB1-2 groups inhibit the Ubiquitin system significantly. All of FB1-2, FB1-3, FB1-4 and FB1-5 groups presented obvious inhibitory effects on Glycosphingolipid biosynthesis. In addition, electron transfer carriers were obviously elevated in FB1-3, FB1-4 and FB1-5 groups. Only in the FB1-6 group did the PPAR signaling pathway, Lipopolysaccharide biosynthesis proteins and Lipopolysaccharide biosynthesis significantly increase, while Cytoskeleton proteins showed a significant decrease (Figure 10).

## 3. Discussion

It has been highly agreeable that microflora in animal gut tracts plays an important role in host health. Many immune and metabolic diseases, such as chronic inflammation, obesity, diabetes, inflammatory bowel disease and atherosclerosis, are likely to be related to the imbalance of gut bacterial flora [3,5,44,45]. Paying more attention to the balance of intestinal flora is more and more urgent. Like other mycotoxins, fumonisin has detrimental effects on the health of human beings and livestock. Many papers have demonstrated that FB1 could increase the ratio of Sa/So, decrease villi length and cause obvious hepatoxicity and nephrotoxicity [20,35,46,47]. To our knowledge, this study is the first to investigate and demonstrate the dose-related effect of FB1 exposure on fecal microbiota in rodent animals. Furthermore, we also intended to observe the effect of low dosage of FB1 that corresponds to the regulatory limit on fecal bacterial flora.

Intestinal microbiota plays a crucial role in human physiology and metabolism due to its multiple functions. In this study, it was observed that FB1 treatments caused many changes in the abundance and composition of gut microflora. Treatment with FB1 significantly decreased the abundance of Alloprevotella (in FB1-3, FB1-4, FB1-5 and FB1-6 groups) and increased Prevotellaceae_NK3B31_group (in FB1-6 group), Rikenellaceae_RC9_gut_group (in FB1-5 and FB1-6 groups) and Parabacteroides (in FB1-5 group). Many research studies had reported that Alloprevotella was associated with the production of short-chain fatty acids (SCFA) and that Alloprevotella mainly produce succinate and acetate, which could protect the intestinal mucosal barrier and inhibit inflammation [48,49,50]. In our results, the abundance of Alloprevotella is negatively correlated with FB1 exposure. Further, it is crucial that we found that the abundance of Alloprevotella in the low dose of FB1 (FB1-1) already showed an obvious decreased tendency, which informed us to pay more attention to the PMTDI of 2 µg/kg bw for FB1, FB2 and FB3, alone or in combination. What is more, our data showed that the dose of FB1-4 below the regulatory limit of 4 mg/kg from USFDA presented a significant decrease of Alloprevotella. This suggested that the regulatory limit from USFDA may be not protective enough. Rikenellaceae has been reported to be related to the pathological progression of inflammatory bowel disease [51] and circadian rhythm disorders, especially in female mice [52]. In our study, we found that Rikenellaceae rose after FB1 treatment. All the evidence indicated that the toxicity of FB1 may be closely related to the chronic inflammation in animals. Consistent with the literature, the dominant phyla of mice were Firmicutes and Bacteroidetes [53]. We determined that exposure of FB1 did not significantly change the abundance of Firmicutes, Bacteroidetes and Proteobacteria. Our results also found that FB1 significantly increased the Cyanobacteria phylum in the FB1-6 group. Little is known about the functions of Cyanobacteria and the influence of Cyanobacteria on animal intestinal microflora [16,54]. Some research found that some species in Cyanobacteria could produce toxic metabolites known as cyanotoxins [55]. Moreover, the toxicity of cyanotoxins is strain-specific [56]. Therefore, positive identification does not predict the hazard level, that is to say, it is difficult to speculate the consequences of the increase of Cyanobacteria caused by FB1, and more studies are needed.

The phylogenetic tree reflects the similarity and difference relationship among different samples. From our phylogenetic tree results, these seven groups have been distinguished well, which indicated that FB1 may present a progressive damage effect on mice along with the increase of doses. Furthermore, the low dosage of FB1 also caused the obvious changes of fecal bacterial flora in mice. It reminded us that we should think more about the long-term accumulation of lower dose of FB1 in body. Additionally, intestinal microbiota is involved in many functional pathways through which the health of host is influenced [57]. In order to estimate how the intestinal microbiota changed after FB1 exposure, the KEGG database was used to compare the sequencing data of these groups. Based on PICRUSt analysis, the results showed that some different pathways were investigated, including the Ubiquitin system, Glycosphingolipid biosynthesis, Electron transfer carriers, PPAR signaling pathway, Lipopolysaccharide biosynthesis proteins, Lipopolysaccharide biosynthesis and Cytoskeleton proteins, etc. From the results, a low dose of FB1 (FB1-1) exposure has caused significant changes in the Ubiquitin system, Selenocompound metabolism, N-glycan biosynthesis, Apoptosis and Meiosis. Ubiquitination is a post-translational modification process, and more and more evidence indicate the importance of ubiquitination in the immune system’s response [58]. Selenium is an essential trace element and is important for various aspects of human health. Moreover, its biological effects are dependent on its incorporation into selenoproteins, which are involved in the modulation of the immune response, inflammatory processes as well as chemoprevention [59,60]. Besides, the N-glycan has influence on the transport of glycosylated proteins, and the N-glycan-dependent enzyme complex may link the processing of N-glycosylated glycosyltransferases with glycosphingolipid metabolism [61], which presented visible alteration in FB1-2, FB1-3, FB1-4 and FB1-5-treated groups. It is reported that glycosphingolipids have specific functional roles essential for survival, proliferation and differentiation during brain development [62], which may be the likely cause of the induction of ELEM by FB1. Further related research is underway by our team. What is more, the high dose of FB1 (FB1-6) exposure caused significant changes in the PPAR signaling pathway, Lipopolysaccharide biosynthesis proteins, Lipopolysaccharide biosynthesis and Cytoskeleton proteins. The pathway of Lipopolysaccharide biosynthesis may cause the formation of numerous lipopolysaccharides (LPS), which is the main constituent of the outer membrane of most of the Gram-negative bacteria and can initiate a strong immune response and serves as an early warning signal of bacterial infection [63,64,65]. From these metabolic profiles analyses, we found that there may be an interesting connection or progressive change from FB1-1 to FB1-6: Ubiquitin system–Glycosphingolipid biosynthesis–Inflammation. All of the above evidence suggested that FB1 exposure could finally induce wide inflammation in mice through influencing many other metabolism pathways.

Since there are few research studies reported that are associated with the effects of FB1 on intestinal bacterial flora in animals such as weaned pigs [39] and broiler chickens [40], more investigations are needed to improve the acknowledgement of FB1.

## 4. Materials and Methods

### 4.1. FB1 Solution Preparation

The FB1 solution was prepared by dissolving FB1 powder (Pribolab, Qingdao, China) in distilled water. First of all, we obtained the 0.4374 mg/mL FB1 solution and then diluted it with distilled water to reach the concentrations of 0.1458 mg/mL, 0.0486 mg/mL, 0.0162 mg/mL, 0.0054 mg/mL and 0.0018 mg/mL.

### 4.2. Animal Trial

In this study, female BALB/c mice (SPF grade, HFK BIOSCIENCE Co., LTD. Beijing, China), 18–20 g body weight with no specific pathogens were used. The mice were acclimated for one week with non-restricted access to commercial feed and water. They were maintained in the environment with 20 ± 3 °C temperature and 50.0 ± 10.0% humidity and a 12 h light/dark cycle. Animal care and all experimental procedures were approved by the Animal Ethics Committee at PUMC&CAMS and were conducted in accordance with the health criteria for the care of laboratory animals enacted by the Beijing municipal government. The mice were then randomly divided into 7 groups, which were labeled as the control, FB1-1 (0.018 mg/kg), FB1-2 (0.054 mg/kg), FB1-3 (0.162 mg/kg), FB1-4 (0.486 mg/kg), FB1-5 (1.458 mg/kg) and FB1-6 (4.374 mg/kg). The dose of FB1-1 corresponds to the PMTDI (provisional maximum tolerable daily intake) of 2 µg/kg bw for FB1, FB2 and FB3, alone or in combination by JECFA (JOINT FAO/WHO EXPERT COMMITTEE ON FOOD ADDITIVES) [66]. In addition, the dose of FB1-4 and FB1-5 crossed over the recommended levels of 4 mg/kg for total fumonisins (FB1 + FB2 + FB3) in whole or partially degermed dry milled corn products (e.g., flaking grits, corn grits, corn meal, corn flour with fat content of >2.25%, dry weight basis) by USFDA [67]. The control group was fed intragastrically with distilled water, while other groups were fed intragastrically with the corresponding FB1 solutions, respectively. The feeding dosage was 10 mL/kg per mouse each time, once a day for 8 weeks.

### 4.3. Sample Collection

Fresh feces were collected from the mice after 8 weeks of exposure to FB1 and then placed in liquid nitrogen at once and stored at −80 °C.

### 4.4. 16S rRNA Gene Sequencing of Fecal Bacterial Flora

The total genomic DNA was extracted from fecal samples by using the PowerSoil DNA Isolation Kit (MoBio Laboratories, Carlsbad, CA, USA) according to the manufacturer’s instructions. The quality and concentrations of DNA were measured by the NanoDrop ND-1000 Spectrophotometer (NanoDrop Technologies, Wilmington, DE, USA). The V3-V4 hypervariable region of the 16S rRNA genes were amplified and purified using the PCR primers with barcode. The sequencing library was quantified by Qubit and qPCR, and the barcoded V3-V4 PCR amplicons were sequenced using the MiSeq platform (Illumina, San Diego, CA, USA) in the double-ended sequencing mode according to the manufacturer’s instructions.

The obtained paired-end reads were merged into tags, and the sequences used in the subsequent analysis were obtained by splicing raw sequence reads using FLASH (Version 1.20) software [68] and eliminating chimeric sequences using USEARCH (Version 10.0.240) software.

### 4.5. Taxonomy Classification and Data Analysis

Sequences with 97% similarity were assigned to the same operational taxonomic unit (OTU) using UPARSE software [69], and taxonomic annotation was conducted using the RDP classifier. The OTU abundance information was normalized using a standard of sequence number corresponding to the sample with the fewest sequences. The alpha diversity was conducted with Quantitative Insights Into Microbial Ecology (QIIME, Version v.1.8.0, http://qiime.org/, accessed on 25 August 2021) software, which is usually used to assess the diversity and abundance of microbes. The unifrac distance was obtained by QIIME (Version 1.8.0, http://qiime.org/, accessed on 25 August 2021) software, and PCA (Principal Component Analysis) was performed using R software. The LEfSe analysis was performed to obtain differences in the abundance, and the threshold of LDA score was 3.0. Besides, in this study, the KEGG database was used to annotate microbiome genes and predict the metabolic function of microbiota with the PICRUSt (Phylogenetic Investigation of Communities by Reconstruction of Unobserved States) program (http://picrust.github.io/picrust/, accessed on 25 August 2021) [70].

### 4.6. Statistical Analysis

Statistical significance was determined following the test using either a one-tailed *t*-test or one-way analysis of variance (ANOVA) with Tukey’s multiple comparison to compare the means of multiple groups. Data are shown as mean ± SEM and were considered statistically significant at *p* < 0.05. GraphPad Prism 7 (GraphPad Software Inc., LaJolla, CA, USA) was used for analysis.

## 5. Conclusions

This study investigated the influences of exposure to different doses of FB1 on fecal bacterial flora in female BALB/c mice. The results indicate that exposure to FB1 in mice shifts the structure and composition of the fecal bacterial community. The apparent changes appear in genus types including Alloprevotella, Prevotellaceae_NK3B31_group, Rikenellaceae_RC9_gut_group, Parabacteroides and phylum types including Cyanobacteria. Moreover, there are some indicated pathways influenced by FB1. To some degree, the minimum dose of FB1 (FB1-1, corresponding to the PMTDI of 2 µg/kg bw for FB1, FB2 and FB3, alone or in combination by JECFA) in our study also presented potential influences on fecal bacterial flora. Subsequently, our team will analyze the changes in biochemical indicators and histopathology to confirm the effects of sequence concentration FB1 in mice. Moreover, the depth of toxic mechanisms and the mechanisms of influence on fecal microbiota are the focus of our next study on FB1.

## Data Availability

Data available on request due to restrictions e.g., privacy or ethical.
